# *I BET* on anti‐FGFR to fight cancer resistance

**DOI:** 10.15252/emmm.201810116

**Published:** 2019-01-04

**Authors:** Rosaria Benedetti, Lucia Altucci

**Affiliations:** ^1^ Department of Precision Medicine University of Campania ‘Luigi Vanvitelli’ Naples Italy

**Keywords:** Cancer

## Abstract

L. Altucci and R. Benedetti discuss the study by Chua *et al* (in this issue of EMBO Molecular Medicine), in which co‐targeting of FGFR signaling increases the responses of metastatic uveal melanoma to BET inhibitors.

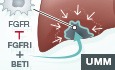

As cancer is a multifactorial disease, the use of and data mining derived from integrated genome and epigenome studies coupled with biochemical, biological, molecular, and epidemiological data is vital to its understanding (Nebbioso *et al*, 2018). Although available therapies lead to regression or improve control of a wide variety of tumors, some do not respond to therapeutic patterns, displaying low survival and high frequency of recurrence. Highly lethal tumors are a group of cancers with (i) an average 5‐year survival rate of about 20% (or less), commonly characterized by frequent late diagnosis (associated with the onset of advanced disease symptoms), and (ii) biological aggressiveness and limited treatment efficacy. Early biomarkers of response and novel therapeutic approaches for these tumor subtypes represent an unmet need. Alternative treatment options include so‐called “targeted therapies”, which inhibit pro‐tumorigenic pathways frequently altered by somatic mutations. Although these therapies are largely efficacious, they are challenged by the development of resistance.

Targeting transcriptional dependencies associated with deregulation of chromatin regulators, transcription factors, and/or cofactors may therefore provide a different strategy (Bradner *et al*, 2017). Inhibition of bromodomain and extraterminal domain (BET) proteins (French, 2016) is emerging as a promising anticancer strategy to block transcriptional dependencies, yet resistance development remains a challenge (Kurimchak *et al*, 2016). Uveal melanoma (UM) is the most common primary intraocular malignancy in adults in the United States, in which roughly 50% of patients develop metastases, predominantly to the liver. Since no therapies have been approved for metastatic UM (UMM), prognosis is very poor, partially as a result of development of resistance to targeted therapies. BET inhibitors (such as PLX51107) are currently being tested in clinical trials for patients with advanced malignancies including UM and UMM, but resistance has been reported.

The present study by Chua *et al* (2019) suggests that inhibition of the FGF receptor (FGFR) pathway improves the response of UMM to BET inhibitors. Specifically, they found that FGF2, but not other growth factors, provides resistance to growth suppression by BET inhibitors in UMM cells as well as *in vivo*. FGF2 effects were reversible by FGFR inhibitors. BET inhibitors also increased FGFR protein expression in UM cells and in patient samples. Interestingly, PLX51107 increased *in vivo* tumor growth of UM cells co‐injected into mice with hematopoietic stem cells, and the combination of PLX51107 and the FGFR inhibitor AZD4547 suppressed tumor growth. Thus, the authors suggest that in patients with UMM, co‐targeting of the FGF2/FGFR cascade is required to improve the efficacy of BET inhibitors, preventing the development of resistance. The finding that liver cells secrete FGF2, crucial to conferring resistance (schematically summarized in Fig 1), is of particular interest, since it provides an example of how to delineate the interaction of organ‐specific cells of the liver with tumor cells. It may indicate that liver microenvironment plays an active role in reducing the efficacy of BET inhibitors, and co‐inhibition of FGFRs by AZD4547 treatment significantly suppresses tumor growth compared to PLX51107‐treated mice.

**Figure 1:**
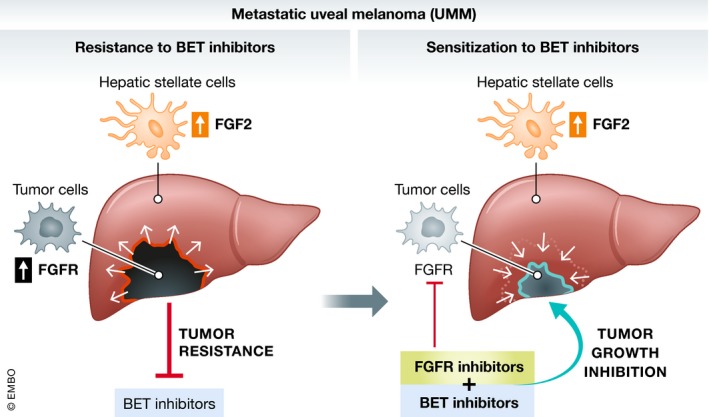
Mechanism of resistance to BET inhibitors in UMM: FGF2 (red) increased production (LSC, yellow) and tumor (black) FGFR‐increased expression leads to BETi resistance (left). Combination treatment BETi+FGFRi suppresses tumor growth (right).

Research approaches of this kind may also be of great importance for future therapies against hepatic metastatic disease. The fact that different mechanism(s) of resistance to BET inhibitors have been reported points to the development of cancer‐specific, and possibly patient‐selective, tumor microenvironment targeting as well as potential specific organ‐context features. This strongly suggests that therapeutic regimens designed to overcome cancer resistance might not only work directly against cancer cells, but likely reset the homeostatic control of cancer development in the whole body. This opens the way toward even more complex personalization in which, depending on the clinical and systemic case, resistance mechanisms might be highly heterogeneous. Intriguingly, different cancer types may share some of these deregulations, suggesting that very diverse cancers might display a common denominator for resistance development. As also described by Chua *et al* (2019), resistance to BET inhibitors in ovarian cancer was associated with elevated expression of FGFRs (Kurimchak *et al*, 2016). The mechanisms underlying BET inhibitor‐induced overexpression of FGFRs are unclear, but may involve modulation of BRD4‐induced regulation of FGFR transcription. BRD4 occupancy was shown at the promoter region of receptor tyrosine kinases and was attenuated by BET inhibitors (Stuhlmiller *et al*, 2016). Thus, although different mechanism(s) for FGFRs are reported, a therapeutic scheme combining BET inhibitors and FGFR inhibition by AZD4547 treatment may be beneficial also against ovarian cancer. All these approaches could, in principle, overcome developed resistance. It remains to be determined whether the application of such strategies might even prevent resistance arising, or would instead act against the development of different mechanisms of relapse.

The study of resistance requires the use of three‐dimensional cell cultures (or immune‐competent *in vivo* models), given the essential role of the tumor microenvironment (and the whole organism), which is not present in cell cultures. This should be further pursued both for a more critical evaluation of the “weight” of studies using only cell cultures and for the choice of disease models. Novel technological developments (such as single‐cell studies and organoid‐like models of disease and treatment resistance) may dramatically modify our understanding of cancer resistance, potentially identifying predispositions, the role(s) of cancer heterogeneity, and “bad/good” actions of immune cells as well as defining, in principle, tailored treatments.

The complex cross‐talk between genome and epigenome deregulations in cancer may play a key role in resistance development. Translating the advances of epigenome deregulation to the clinic may thus require a better understanding of the anticancer action of so‐called epigenetic drugs (epidrugs), for example, in making the choice between targeting transcriptional dependencies (associated with deregulation of chromatin regulators such as BET) and/or using therapeutic strategies against mutated chromatin players. It is tempting to speculate that different epigenome targeting may, in the future, lead to the use of “pure” epigenome player‐targeted treatments of cancer and cancer resistance. For example, since histone deacetylase inhibitors (HDACi) are reported to reverse FGF2‐induced growth (Lee *et al*, 2018), an HDACi‐dependent approach coupled with BET inhibitors could hypothetically represent an alternative strategy to overcome (or prevent?) resistance development in UMM. Vorinostat (a well‐known HDACi) is entering a Phase I and II clinical trials for UMM (NCT00121225 and NCT01587352) (Moschos *et al*, 2018) (Haas *et al*, 2014), and in the near future, the potential of epigenome‐targeting schemes will likely be better defined.
